# Evaluation of Poly(Lactic-co-glycolic) Acid Alone or in Combination with Hydroxyapatite on Human-Periosteal Cells Bone Differentiation and in Sinus Lift Treatment

**DOI:** 10.3390/molecules22122109

**Published:** 2017-12-02

**Authors:** Gabriele Ceccarelli, Rossella Presta, Saturnino Marco Lupi, Nefele Giarratana, Nora Bloise, Laura Benedetti, Maria Gabriella Cusella De Angelis, Ruggero Rodriguez y Baena

**Affiliations:** 1 Department of Public Health, Experimental Medicine and Forensic, Human Anatomy Unit, University of Pavia, 27100 Pavia, Italy; laura.benedetti@unipv.it (L.B.); cusella@unipv.it (M.G.C.D.A.); 2 Center for Health Technologies, University of Pavia, 27100 Pavia, Italy; nora.bloise@unipv.it; 3 Department of Clinico-Surgical, Diagnostic and Pediatric Sciences, School of Dentistry, University of Pavia, P.le Golgi 2, 27100 Pavia, Italy; rossella91ta@gmail.com (R.P.); saturnino.marco.lupi@gmail.com (S.M.L.); ruggero.rodriguez@unipv.it (R.R.y.B.); 4 Department of Development and Regeneration, Laboratory of Translational Cardiomyology, KU Leuven, B-3000 Leuven, Belgium; nefele.giarratana@studenti.unich.it; 5 Molecular Medicine Department (DMM), Center for Health Technologies (CHT), UdR INSTM, University of Pavia, Viale Taramelli 3/B, 27100 Pavia, Italy; 6 Department of Occupational Medicine, Toxicology and Environmental Risks, Istituti Clinici Scientifici Maugeri S.p.A, IRCCS, Via S. Boezio 28, 27100 Pavia, Italy

**Keywords:** tissue engineering, periosteum-derived stem cells, poly(lactic-co-glycolic) acid-based materials, osteoconductivity

## Abstract

Most recent advances in tissue engineering in the fields of oral surgery and dentistry have aimed to restore hard and soft tissues. Further improvement of these therapies may involve more biological approaches and the use of dental tissue stem cells in combination with inorganic/organic scaffolds. In this study, we analyzed the osteoconductivity of two different inorganic scaffolds based on poly (lactic-co-glycolic) acid alone (PLGA-Fisiograft) or in combination with hydroxyapatite (PLGA/HA-Alos) in comparison with an organic material based on equine collagen (PARASORB Sombrero) both in vitro and in vivo. We developed a simple in vitro model in which periosteum-derived stem cells were grown in contact with chips of these scaffolds to mimic bone mineralization. The viability of cells and material osteoconductivity were evaluated by osteogenic gene expression and histological analyses at different time points. In addition, the capacity of scaffolds to improve bone healing in sinus lift was examined. Our results demonstrated that the osteoconductivity of PLGA/HA-Alos and the efficacy of scaffolds in promoting bone healing in the sinus lift were increased. Thus, new clinical approaches in sinus lift follow-up should be considered to elucidate the clinical potential of these two PLGA-based materials in dentistry.

## 1. Introduction

Tissue engineered-based approaches represent an important challenge in craniofacial reconstruction [1,2,3]. In fact, the main limitation of maxillofacial surgery is the ability to achieve the regeneration of hard and soft tissues lost in cases of trauma, disease, or medical issues. The combination of cells, scaffolds, and growth factors is known as the “tissue engineering triad” [4,5,6,7], making up the key components of engineered biocomplexes. For this reason, elucidation of the molecular interactions among scaffolds, stem cells, and the in situ microenvironment remains the main objective in regenerative medicine and dentistry.

Different groups have described the positive association between scaffolds and stem cells in maxillofacial and dental bone regeneration [8,9,10,11]. With regard to stem cells, periosteum-derived progenitor cells (PCs) display mesenchymal stem cell (MSC) properties, such as the capacity to differentiate into mesodermal tissues, which contribute to matrix organization and bone architecture [12,13]. Several studies have demonstrated that human periosteal cells (hPCs) represent a promising source for cell-based osteoinductive grafts in oral surgery, not only with regard to the ease of collection but also for the rapid in situ engraftment [13,14]. Among the biomaterials used in dentistry, polylactic–polyglycolic acid (PLGA-Fisiograft) is a common copolymer obtained by the union of lactic and glycolic acid through ester bonds [6,15,16]. The final composition of the polymer chain influences the degradation time, prolonging the half-life of the material in the oral cavity once applied in situ. This bone substitute is used extensively for bone regeneration in dentistry and has been combined with growth factors or inorganic materials and MSCs with promising results [17]. Its versatility it is also due to the variety of available forms, including hydrogels, microspheres, blocks, and fibers [18]. Recent developments have highlighted the potential of porous hydroxyapatite (HA) as a synthetic bone graft [19,20]. HA exhibits a strong propensity for attracting osteoblasts. In fact, its chemical composition is very similar to the mineral component of the mammalian bone [21], but possesses a low resorption rate in vivo and is brittle, particularly in highly porous forms [22]. The addition of biodegradable PLGA to HA (PLGA/HA-Alos) would allow for better manipulation and biocompatibility and would permit the creation of biocomplexes with stem cells more able to fit bone defects. Similar to inorganic materials, collagen-based scaffolds, such as PARASORB Sombrero (RESORBA Medical GmbH), are also used in dentistry. This specific collagen matrix consists of a membrane-cone made of equine collagen, and its applications include socket preservation, treatment of the alveolus, and other bone defects [23].

In this study, we first present an in vitro analysis of periosteal cells grown in contact with PLGA/HA-Alos (Allmed s.r.l., Lissone (MB), Italy), PLGA-Fisiograft (Ghimas s.p.a., Casalecchio di Reno (BO), Italy), and PARASORB Sombrero chips, with the aim of comparing the osteoinductive potential of these materials in comparison with hPCs seeded on plastic. Our cellular samples were isolated from different patients that underwent oral surgery for various reasons. We then present clinical results of PLGA/HA-Alos and PLGA-Fisiograft in the sinus lift in order to validate the in vitro results with clinical applications.

## 2. Results

### 2.1. Effects of Scaffolds on the Proliferation of hPCs

**Figure 1:**
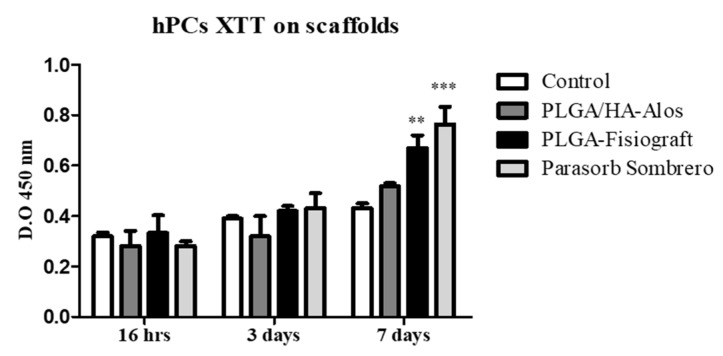
XTT test on hPC cells growth in contact or not with chips of PLGA/HA-Alos, PLGA-Fisiograft and PARASORB Sombrero at 16 h, 3 and 7 days of culture in proliferative medium. **: *p* < 0.01, ***: *p* < 0.001 for PLGA/HA-Fisiograft and PARASORB Sombrero versus the control.

hPCs cultured in contact with chips of PLGA-Fisiograft, PLGA/HA-Alos, or PARASORB Sombrero at seven days displayed higher viability measurement with respect to cells seeded on plastic (control). Figure 1 shows a bar graph considering three time points: 16 h after plating, three and seven days of hPC cell culture in proliferative medium. These results demonstrate that hPCs growth was promoted by the presence of biomaterials. This enhancement is more evident at seven days especially for PLGA-Fisiograft and PARASORB Sombrero. The small difference of OD between all samples at 16 h and three days is probably due to the longer doubling time of these primary cells of human origin (about 76 h).

### 2.2. Gene Expression Analysis

**Figure 2:**
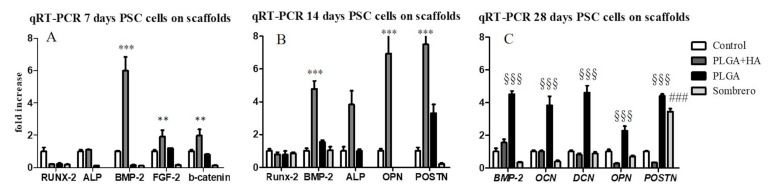
Expression of the indicated bone-specific markers as determined by qRT-PCR. hPCs were seeded and cultured in contact with PLGA/HA-Alos, PLGA-Fisiograft, and PARASORB Sombrero for 7 (panel **A**), 14 (panel **B**), or 28 days (panel **C**). The graph shows the fold induction of gene expression expressed in arbitrary units, with the expression of genes in cells grown on plastic set as 1. **: *p* < 0.01 for PLGA/Ha-Alos, ***: *p* < 0.001 for PLGA/HA-Alos versus the control; §§§: *p* < 0.001 for PLGA-Fisiograft versus the control; ###: *p* < 0.001 for PARASORB Sombrero versus the control.

In order to characterize the cell genotype, gene expression analysis was performed in hPCs cultivated in PM in contact with the three different scaffolds. Quantitative real-time reverse transcription polymerase chain reaction (qRT-PCR) was performed at 7, 14, and 28 days of culture (Figure 2). Table 1 shows the primers used for qRT-PCR. Figure 2 shows the fold induction of the investigated genes expressed in arbitrary units calculated based on the expression of genes in cells grown on plastic, which was set to one (control cells). After seven days of culture, hPCs seeded in contact with PLGA/HA-Alos showed strong up-regulation of bone morphogenic protein (*BMP*)-2 (5-fold increase; *p* < 0.001) and minimal increase in alkaline phosphatase (*ALP*; 0.3-fold increase), fibroblast growth factor (*FGF*)-2 (1.5-fold increase), and β-catenin (1-fold increase) expression compared with control cells. Intriguingly, after 14 days of culture (Figure 2B), *BMP*-2 up-regulation was maintained for cells seeded in contact with PLGA/HA-Alos (4-fold increase; *p* < 0.05), accompanied by up-regulation of *ALP* (3-fold increase; *p* < 0.05), osteopontin (*OPN*; 6-fold increase; *p* < 0.001), and periostin (*POSTN*; *p* < 0.001). The same genes showed no remarkable differences in expression in cells cultured in contact with PLGA-Fisiograft and PARASORB Sombrero at these time points (Figure 2A,B). These results indicated that the scaffold PLGA/HA-Alos had strong osteoinductive effects on hPCs at 7 and 14 days of culture. At 28 days, osteocalcin (*OCN*), *OPN*, *POSTN*, *BMP-2*, and decorin (*DCN*) genes were up-regulated (3-fold increase; *p* < 0.001) in cells seeded in contact with PLGA-Fisiograft compared with control cells and cells grown on PLGA/HA-Alos and PARASORB Sombrero. Taken together, these results indicated that, even in proliferative medium, the bone genotype program was enhanced and accelerated in hPCs cultivated in contact with PLGA/HA-Alos chips compared with cells seeded on plastic or in contact with the other scaffolds. Moreover, chips of PLGA without hydroxyapatite (HA; Fisiograft) induced similar osteogenic effects in hPCs, albeit at the end of the culture period (28 days, Figure 2C).

### 2.3. Morphological Evaluation of Calcium Deposition

**Figure 3:**
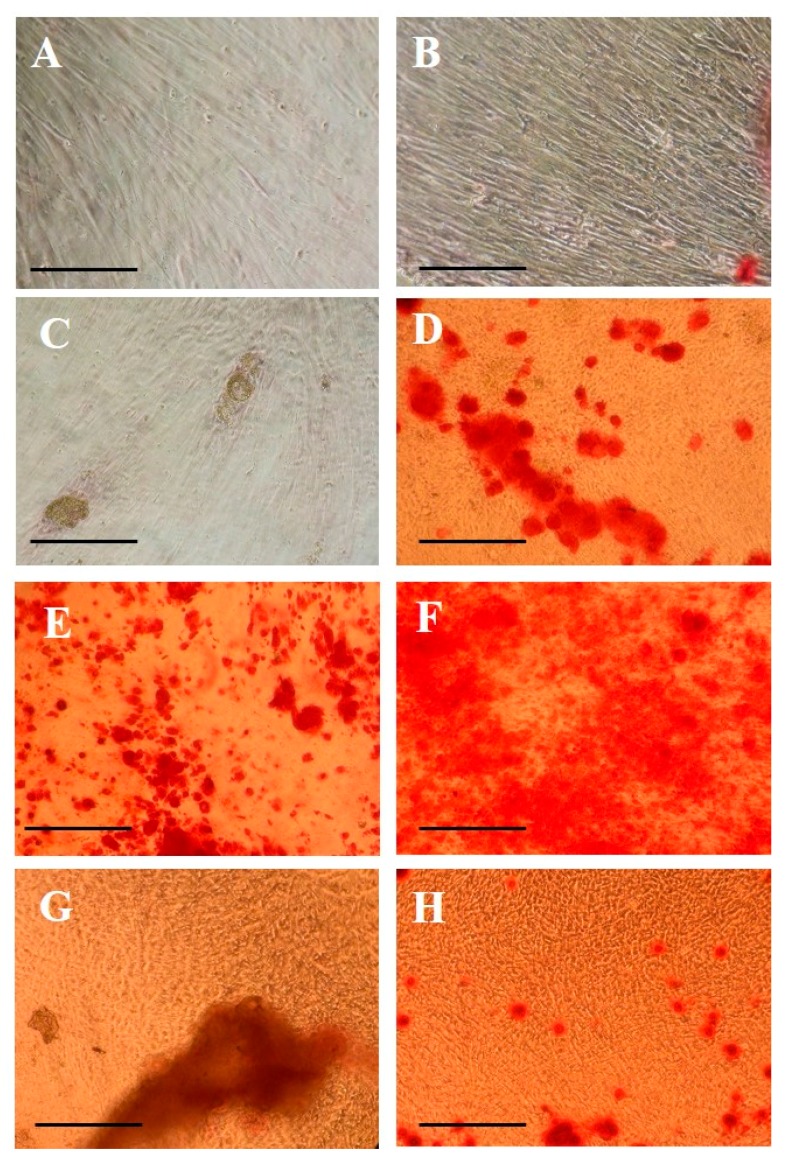
Alizarin Red S staining results in hPCs after 14 and 20 days of culture. (**A**,**B**) Control hPCs at 14 and 20 days; (**C**,**D**) hPCs in contact with PLGA-Fisiograft at 14 and 20 days; (**E**,**F**) hPCs in contact with PLGA/HA-Alos at 14 and 20 days; (**G**,**H**) hPCs in contact with PARASORB Sombrero at 14 and 20 days. All images are at 10× magnification. The scale bars are equivalent to 50 μm.

Morphological studies (Alizarin Red S staining) performed at 14 and 20 days of culture of hPCs grown in contact with scaffolds are shown in Figure 3 (10× magnification). In Alizarin Red-stained hPCs at 14 days seeded in contact with PLGA/HA-Alos, the presence of mineralized nodules was higher than that in cells grown in contact with other scaffolds or the control (Figure 3E). This effect was maintained and increased at 20 days (Figure 3F). Interestingly, at 20 days, hPCs grown in contact with PLGA-Fisiograft and PARASORB Sombrero began to show increased calcium nodules compared with cells grown on plastic (Figure 3D,H), consistent with the gene expression results. In fact, data at 28 days show that the expression of osteogenic genes was higher in cells grown on PLGA-Fisiograft than control cells. The positive effects of the combination of scaffolds were evident, indicating their differential osteoconductive properties on hPC cell differentiation.

### 2.4. Bone Matrix Deposition: Quantification and Immunolocalization Analysis

In order to evaluate the amount of extracellular matrix constituents produced by hPCs seeded in contact with the three materials in PM, enzyme-linked immunosorbent assays (ELISAs) were performed after 28 days of culture. Table 2 shows the protein content results.

There was a significant enhancement in the deposition of all osteogenic proteins in cells cultivated in contact with PLGA/HA-Alos compared with that in hPCs seeded on plastic, PLGA-Fisiograft, and PARASORB Sombrero (Table 2). For the other materials, no differences were observed in protein extracellular matrix (ECM) content, particularly for PARASORB Sombrero, whereas a slight increase was observed in protein content in cells grown in contact with PLGA-Fisiograft in comparison with control cells. These results were similar to the results of gene expression analysis (Figure 1), highlighting that PLGA/HA-Alos appeared to be more osteoinductive than the other materials tested.

### 2.5. Clinical Results

**Figure 4:**
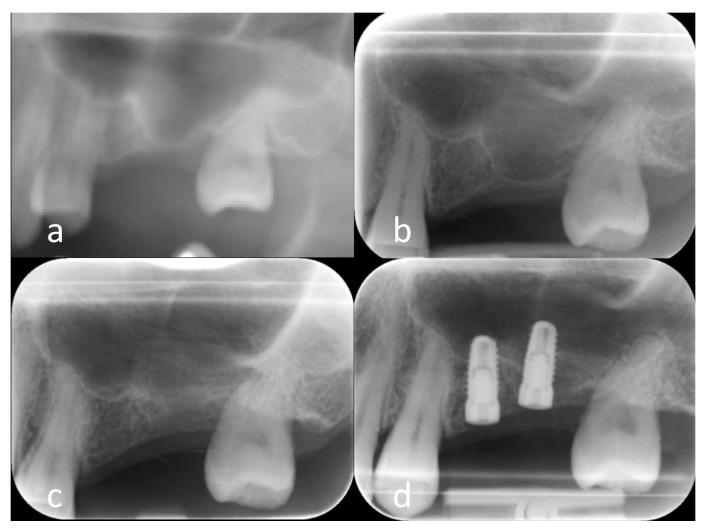
PLGA group. (**a**) Magnification of panoramic radiograph of left sinus showing a residual bone height insufficient to implant positioning; (**b**) periapical X-ray of the same site of (**a**) immediately after sinus floor elevation surgery; (**c**) periapical X-ray of the same site of (**a**) six month after sinus floor elevation surgery; (**d**) periapical X-ray after implant positioning in the new-formed bone.

**Figure 5:**
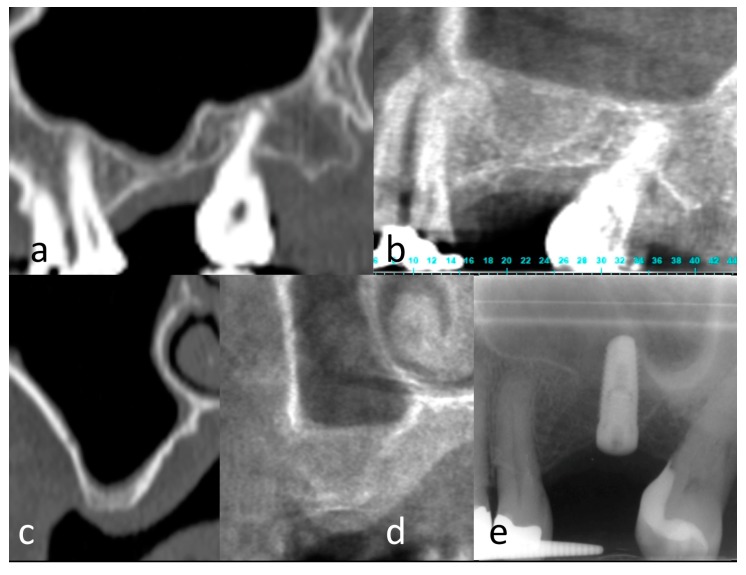
PLGA/HA group. (**a**,**c**) Computed Tomography of left sinus showing a residual bone height insufficient to implant positioning in panoramic and cross-sectional view; (**b**,**d**) Cone Bean Computed Tomography of the same site of (**a**,**c**) six months after sinus floor elevation surgery; (**e**) Periapical X-ray after implant positioning in the new-formed bone.

A total of 10 sinuses from nine patients (mean age: 52 ± 10 years) were grafted, including five in the PLGA-Fisiograft group and five in the PLGA/HA-Alos group. In all cases, after a six-month healing period, correct implant placement with good implant stability was achieved, and the grafts were considered successful (Figure 4 and Figure 5). In the PLGA/HA-Alos group, a total of eight implants were placed (mean: 1.6 ± 0.5 implants/patient), and in the PLGA-Fisiograft group, seven implants were placed (mean: 1.4 ± 0.5 implants/patient). The mean vertical radiographic increase for the PLGA/HA-Alos group was 8.8 ± 3.0 mm, whereas that for the PLGA-Fisiograft group was 8.2 ± 3.5 mm (difference not significant; *p* = 0.52). Interestingly, PLGA-Fisiograft grafts appeared more radiolucent than PLGA/HA-Alos grafts.

## 3. Discussion

The most important purpose of tissue engineering is to create therapeutic substitutes for regenerating tissues and organs. In dentistry and maxillofacial surgery, the reconstruction of critical-size mandibular or alveolar bony defects remains a challenge; thus, bone regeneration using cell-seeded scaffolds has been investigated [1,2,3,4,5,6]. The ideal requirements that a scaffold should have to improve bone healing are high porosity and an adequate pore size to facilitate cell seeding and diffusion of cells and nutrients; the capacity to transport nutrients, oxygen, and metabolites. Other important characteristics should be: Biodegradability, since scaffolds need to be absorbed by the surrounding tissues without the necessity of surgical removal; biocompatibility and adequate physical and mechanical strength [24,25]. In dentistry, the combination between scaffolds and stem cells remains a key challenge for bone and tissue healing. Several authors have demonstrated the efficacy of MSCs seeded on different types of scaffolds (calcium phosphate cement [CPC], magnesium phosphate cement (MPC), and a calcium-MPC (CMPC) in the maxillary sinus floor in rabbits [26]. Moreover, researchers have shown that CMPC can better facilitate new bone formation and mineralization than CPC or MPC and that the addition of MSCs could further promote its osteogenic capacity [27]. A previous study [28] demonstrated the ability of micrograft in conjunction to PLGA–HA to promote bone formation. The present study is aimed to compare different anorganic (PLGA, PLGA–HA) and organic (collagen) substrates to individuate which are the most suitable for bone regeneration in conjunction to autologous micrograft.

In this manuscript, we performed in vitro studies to assess the efficacy of inorganic and organic scaffolds to promote periosteal cells bone differentiation, comparing the use of the same materials for bone healing in patients undergoing sinus augmentation. The inorganic scaffolds used in this study were based on PLGA alone or in combination with HA. Several studies have demonstrated that PLGA-Fisiograft is preferred compared with its constituent homopolymers for the fabrication of bone substitute constructs, although the clinical applications of this scaffold are limited by its osteoconductivity. Therefore, the scaffold was combined with HA, an inorganic material largely used in bone tissue engineering due to its nontoxicity, bioactivity, and osteoconductivity and its similarity to bone ECM (PLGA/Ha-Alos). Concurrent with our analysis of these inorganic materials, we tested the organic material PARASORB Sombrero, a membrane-cone made with equine collagen in in vitro experiments. We analyzed in vitro the proliferation and bone differentiation of hPCs grown in contact with PLGA-Alos, PLGA-Fisiograft, and PARASORB Sombrero at different time points during 28 days of culture in PM. We also performed clinical analyses in the sinus lift to compare PLGA-Fisiograft and PLGA-Alos in clinical applications.

Our in vitro tests focused on the osteoconductive capacity of biomaterials in hPCs isolated from patients who underwent periodontal surgeries. First, we found that chips of organic/inorganic scaffolds did not negatively influence hPCs viability. Subsequently, we showed that osteogenic induction of PLGA/HA-Alos occurred rapidly, as visualized by Alizarin Red S staining at 14 days; hPCs displayed more intense red-orange staining of mineralized bone matrix compared with other culture conditions at the same time. This phenomenon was confirmed by the strong gene induction of bone-related genes in cells grown in contact with PLGA/HA-Alos at 14 days. In order to elucidate the early response of hPCs grown in contact with PLGA/HA-Alos chips, we investigated genes related to early and intermediate bone development after seven days of culture. Indeed, *FGF-2* and β-catenin are involved in osteoblast maturation through the Cbfa-1/runt-related transcription factor (*RUNX*)-2 pathway [29]. In particular, several authors demonstrated that β-catenin participates in the process of maturation from pre-osteoblasts to immature osteoblasts in a pathway together with Osterix and *RUNX-2*, blocking the possible chondrogenic lineage of the mesenchymal progenitor [30]. Therefore, we speculate that at seven days, PLGA/HA-Alos chips would induce strong bone genotype activation via *BMP-2*, *FGF-2*, and β-catenin pathways compared with that in hPCs grown in contact with PLGA-Fisiograft alone, PARASORB Sombrero, and control cells. In addition, the evaluation of ECM deposition by hPCs (28 days) confirmed that PLGA/HA-Alos chips were more osteoinductive compared with the other materials tested. Long-term culture (20 and 28 days) of hPCcells with biomaterials in proliferative medium seems to be anyway sufficient to induce bone differentiation compared with cells grown on plastic, as demonstrated by gene expression studies and morphological tests.

In our in vivo pilot study, bone regeneration in sinus augmentation was performed using only PLGA-Fisiograft and PLGA/HA-Alos because of the inadequate indication of PARASORB Sombrero for this procedure. The clinical results showed good bone regeneration of all biomaterials tested and effective osteointegration of implants located in the regenerated site at the selected healing time. Therefore, larger studies aimed at evaluating the behaviors of PLGA-Fisiograft and PLGA/HA-Alos grafts enriched with hPCs could be conducted in accordance with the presented surgical protocol. Moreover, these clinical cases supported the suitability of each of these bone substitutes mixed together with hPCs, ensuring their biocompatibility and osteointegration in vivo. Interestingly, PLGA-Fisiograft radiographic results were more radiolucent than PLGA/HA-Alos results after six months of healing. This result could be explained by the absence of HA, which is a radiopaque material. However, the clinical results showed no differences between the two groups in terms of graft and implant success. In two-stage procedures, implant surgery was performed 6 months after grafting with no differences between the two groups. In accordance with the in vitro results of this study, further in vivo studies should evaluate whether one or both materials can yield suitable results with shorter healing times. Moreover, long-term follow-up studies should evaluate the differences in radiological aspects of the grafts.

In conclusion, we demonstrated the biocompatibility of the scaffolds and the osteoconductivity of PLGA/HA-Alos in accelerating bone responses in hPCs. Nevertheless, analysis of patients at six months did not confirm the results of our in vitro studies, although good bone restoration in sinus lift treatment was observed. Therefore, new clinical approaches in sinus lift follow-up should be considered, probably at one month, in order to elucidate the clinical potential of the two PLGA-based materials.

## 4. Materials and Methods

### 4.1. Scaffold Composition

Fisiograft (Ghimas SpA, Casalecchio di Reno, Bologna, Italy) is an alloplastic biomaterial formed by 50:50 PLGA. This material is completely bioabsorbable within 6 months and is available in three clinical forms: sponge, granular, and gel. For this study, the sponge form was used. Its clinical effectiveness has been demonstrated, and its clinical indications are related to its function as a space maintainer, namely, socket preservation, maxillary sinus elevation, correction of perimplant and periodontal bone defects, and treatment of dehiscence and fenestrations [31,32].

Alos (Allmed, Lissone, Monza-Brianza, Italy) is an alloplastic biomaterial that is composed of a copolymer of PLGA enriched with about 20% nonsintered porous HA (PLGA/HA). It is completely bioabsorbable within 8 months and is available in two clinical forms: sponge or gel. The sponge form was used for this study. Like PLGA, PLGA/HA-Alos is indicated for clinical use owing to its space maintainer function, e.g., in sinus floor elevation, socket preservation, split crest, and filling of periodontal bone defects or cyst and tumor outcomes [11,33,34,35,36].

PARASORB Sombrero (RESORBA Medical, Nürnberg, Germany) is a xenomaterial and is a membrane-cone made of equine collagen. It is hydrophilic and biocompatible and does not give rise to the inflammatory response, but also degrades in a relative short time and shows a poor load resistance; therefore, its indications are limited and include socket preservation, postextraction site hemostasis, and the filling of limited bone defects as dehiscence and fenestrations. This scaffold is the only nonsynthetic scaffold, and there was therefore an extremely remote possibility of cross-contamination and intolerance reactions [37].

### 4.2. Periosteum-Derived Mesenchymal Cell Isolation and Characterization

Periosteum samples were obtained from four healthy (ASA 0–1) patients that underwent periodontal surgeries at the Department of Clinico-Surgical, Diagnostic and Pediatric Sciences of the University of Pavia. All patients signed informed consent for participation in the study.

During surgery and under local anesthesia, a periosteum sample of 0.5–1 cm^2^ for each patient was harvested with the aid of a disposable sterile blade (15c). The excision of tissue samples did not cause any increase in morbidity or risk for the patient. The freshly harvested sample was washed with sterile physiological solution, inserted into a labeled and anonymous test tube, and then shipped to the laboratory. The tubes contained 5 mL of physiologic sterile saline solution enriched with antibiotics. Subsequently, samples were kept refrigerated at 4 °C before being processed within 24 h. hPCs were isolated by a method previously described [12] and cultivated in α-MEM supplemented with 20% fetal bovine serum (FBS), 100 µM 2-p ascorbic acid, 2 mM l-glutamine, 100 U/mL penicillin, and 1000 mg/mL streptomycin. hPCs were then characterized by fluorescence-assisted cell sorting (FACS) analysis for the following mesenchymal surface antigens: CD34, CD117, CD45, CD90, CD14, CD73, HLA-DR, HLA-ABC (all from BD Bioscience, Buccinasco, Italy), CD105, and CD29 (AbD Serotec, Kidlington, Oxford, UK). hPC cells displayed all mesenchymal markers specific for MSCs [38].

### 4.3. Attachment of hPCs to Scaffolds

To assess the effects of PLGA-Fisiograft, PLGA/HA-Alos, and PARASORB Sombrero to promote in vitro osteoblastic cell differentiation, we seeded 5000 hPCs/cm^2^ on 24-well culture plates to form a confluent monolayer, in contact with chips measuring an average of 0.5 × 2 mm of these different materials. Cells were cultivated in contact with scaffolds for 7, 14, 20 and 28 days in PM (α-MEM plus 20% FBS), changed twice a week. In order to prevent contamination, composite chips were sterilized under ultraviolet light prior to cell seeding. After 24 h of incubation, cells grown in contact with these different types of chips were covered with an agar top, in order to mimic the closure of the surgical site in bone alveolar restoration.

### 4.4. Cell Viability Assay

To evaluate the proliferation of hPCs grown in contact with the three different biomaterials (PLGA-Fisiograft, PLGA/HA-Alos, and PARASORB Sombrero), we performed XTT tests (Sigma Aldrich, St. Louis, MO, USA) at 7 days of culture [39]. The XTT reagent (Roche, Basel, Switzerland) was added to each well, and the plates were incubated for 4 h. A Nanodrop device was then used to read light absorbance at 450 nm. The OD was measured as proportional to the metabolic activity of the cells.

### 4.5. Gene Expression Analysis

Total RNA from hPCs seeded in contact with PLGA-Fisiograft, PLGA/HA-Alos, PARASORB Sombrero, or plastic for 7, 14, and 28 days in PM was extracted and retrotranscribed into cDNA as previously reported [40]. Gene expression analyses were performed by qRT-PCR (Bio-Rad, Mini-Opticon Real-Time PCR System; Bio-Rad, version 1.5, Hercules, CA, USA) using oligonucleotide primers (Table 1). For each time point, we analyzed the expression of different osteogenic genes, including *RUNX-2*, *BMP-2*, *BMP-4* and *ALP* at 7 days; *RUNX-2*, *BMP-2*, *POSTN*, *ALP* and *OPN* at 14 days; and *BMP-2*, *OCN*, *DCN*, *OPN* and *POSTN* at 28 days. The fold expression of each sample was normalized to the expression of glyceraldehyde phosphate dehydrogenase (*GAPDH*) as a housekeeping gene and analyzed in triplicate. The fold increase values were calculated using CFX Manager software (Bio-Rad) with the ΔCt method.

### 4.6. Bone ECM Protein Extraction and ELISAs

Evaluation of ECM proteins produced by cells seeded in contact with PLGA-Fisiograft, PLGA/HA-Alos, PARASORB Sombrero, or plastic dishes in PM was performed at 28 days using ELISAs, as previously reported [41]. The total protein concentration was evaluated with a BCA Protein Assay Kit (Pierce Biotechnology, Inc., Rockford, IL, USA). The total protein concentrations were 165 µg/mL for control hPCs, 275 µg/mL for hPCs grown in contact with PLGA/HA-Alos, 147 µg/mL for hPCs grown in contact with PLGA-Fisiograft, and 90 µg/mL for hPCs grown in contact with PARASORB Sombrero.

### 4.7. Alizarin Red S Test

The Alizarin Red test was used to determine the presence of calcium deposition, an indicator of the osteogenic differentiation [42]. hPCs grown in contact with different scaffolds were stained at 14 and 20 days with pH-adjusted (4.2) 2% Alizarin Red S solution (Electron Microscopy Sciences, Fort Washington, MD, USA), washed, and then photographed using a transmission light microscope (Eclipse E800, Nikon, Konan, Minato-ku, Tokyo, Japan).

### 4.8. Patients

**Figure 6:**
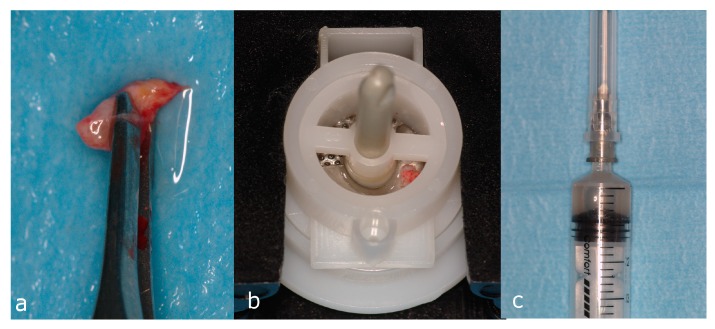
Micrograft suspension preparation in accordance with the Rigenera protocol. A connective tissue sample about 5 mm in length was collected directly from the surgical recipient site and washed with sterile saline (**a**); the sample was inserted in the Rigenera filter (**b**) to obtain the cellular graft enriched with hPCs (**c**).

The in vivo study was conducted at the Department of Clinico-Surgical, Diagnostic and Pediatric Sciences, University of Pavia, Italy (Minutes March 2014 of the Ethic Committee—University of Pavia). Selection criteria were previously reported [33]. Briefly, after obtaining informed consent, patients more than 18 years of age requiring monolateral or bilateral maxillary sinus floor augmentation without comorbid disease contraindicating the procedure (ASA scores 1 and 2) were randomized by coin toss to two groups that differed only in the type of graft used. For the in vivo study, only PLGA-Fisiograft and PLGA/HA-Alos were used due to the unsuitability of PARASORB Sombrero to the sinus augmentation procedure. All patients received a lateral-approach maxillary sinus floor augmentation [43]. When the residual bone height permitted concomitant implant positioning, implants were placed during the same surgery. When a two-step technique was indicated, patients received sinus floor augmentation during the first surgery and then implant positioning and bone sampling from the implant site 6 months later. Sinus floor augmentation with or without immediate implant positioning is an acceptable surgical procedure [11,43,44]. The grafts consisted of randomized biomaterial added to autologous micrografts obtained by mechanical disaggregation of a small portion of cartilage tissue (Rigenera protocol, Figure 6) [45]. In one-stage implant positioning, the grafting procedure was considered successful when radiographic control exhibited a healthy appearance and when implants were stable at the uncovering after 6 months. In two-stage implant positioning, the grafting procedure was considered successful when the bone height was sufficient to correctly place the implant after healing for 6 months.

### 4.9. Statistics

Each experiment was performed in triplicate and in at least three separate experiments. Results are expressed as the mean ± standard deviation. Statistical significance between groups was evaluated by one-way analysis of variance with post-hoc Bonferroni tests, particularly for proliferation and gene expression analysis.

## Figures and Tables

**Table 1:** List of the primers used for qRT-PCR.

Genes	Accession Number	FW	RW	T° Annealing
*ALP*	NM_000478.5	5′-CTATCCTGGCTCCGTGTCC-3′	5′-AGCCCAGAGATGCAATCG-3′	60°
*FGF-2*	NM_002006.4	5′-CGGCTGTACTGCAAAAACGG-3′	5′-TTGTAGCTTGATGTGGAGGGTCG-3′	60°
*RUNX-2*	NM_001278478.1	5′-ACAGTAGATGGACCTCGGGA-3′	5′-ATACTGGGATGAGGAATGCG-3′	60°
*OPN*	NM_001040058.1	5′-GTGATTTGCTTTTGCCTCCT-3′	5′-GCCACAGCATCTGGGTATTT-3′	60°
*OCN*	NM_199173.5	5′-AAGAGACCCAGGCGCTACCT-3′	5′-AACTCGTCACAGTCCGGATTG-3′	60°
*BMP-2*	NM_001200.3	5′-CCTCCGTGGGGATAGAACTT-3′	5′-CACTGTGCGCAGCTTCC-3′	60°
*POSTN*	NM_006475.2	5′-GAGGTCACCAAGGTCACCAAA-3′	5′-GGGTGTGTCTCCCTGAAGC-3′	60°
*DCN*	NM_001920.4	5′-ACCCCCTCCTCCTTTCCACACC-3′	5′-ACCAGGGAACCTTTTAATCCGGGAA-3′	60°
*β-Catenin*	NM_001098209.1	5′-GTCTGAGGAGCAGCTTCAGT-3′	5′-CCATTGTCCACGCTGGATTT-3′	60°
* GAPDH	NM_002046.5	5′-AGCCTCAAGATCATCAGCAATGCC-3′	5′-TGTGGTCATGAGTCCTTCCACGAT-3′	60°

*: Housekeeping gene.

**Table 2:** Protein titration of bone extracellular matrix produced by hPCs cultured for 28 days in a proliferative medium on plastic, PLGA-Fisiograft, PLGA/HA-Alos, and PARASORB Sombrero. Results are expressed as protein quantity (pg)/2 µg and are presented as an average of three measurements from two separate experiments.

Control		PLGA (Fisiograft^®^)		PLGA + HA (Alos^®^)		Parasorb Sombrer^®^	
	pg	pg	Retio/Related to Control	pg	Retio/Related to Control	pg	Retio/Related to Control
ALP	6.59 ± 2.05	10.09 ± 1.60	1.53 *	11.29 ± 2.2	1.71 ***	10.20 ± 3.1	1.5
OSN	1.20 ± 0.27	1.81 ± 1.29	1.5 *	2.16 ± 0.23	1.8 ***	1.75 ± 0.4	1.45
OPN	5.73 ± 1.13	8.76 ± 0.69	1.52 *	15.80 ± 3.6	2.75 ***	9.6 ± 1.12	1.67 *
BMP-2	0	0		123	123	0	
OSC	419 ± 7.81	586 ± 30.12	1.39 *	643 ± 24.2	1.53 ***	352.50 ± 40.25	0.84
DCN	36.24 ± 5.20	38.56 ± 2.5	1.06 *	55.44 ± 3.4	1.52 ***	37.50 ± 10.63	1.03
Type-I-collagen	65.9 ± 3.24	27.6 ± 3.6	0.4 *	73.8 ± 8.7	1.11 ***	22.2 ± 5.21	0.33

* *p* < 0.05; *** *p* < 0.001.

## Abbreviations

The following abbreviations are used in this manuscript:

*ALP*	Alkaline phosphatase
α-MEM	Alpha-minimum essential medium
BCA	Bicinchoninic acid (assay)
β-cat	Βeta-catenin
*BMP*	Bone morphogenetic protein
*FGF-2*	Fibroblast growth factor 2
*DCN*	Decorin
DNA	Deoxyribonucleic acid
ECM	Extracellular matrix
ELISA	Enzyme-linked immunosorbent assay
FACS	Fluorescence-activated cell sorting
GAPDH	Glyceraldehyde phosphate dehydrogenase
HA	Hydroxyapatite
hMSCs	Human mesenchymal stem cells
hPCs	Human periosteal cells
mRNA	Messenger RNA
*OCN*	Osteocalcin
*OPN*	Osteopontin
PLGA	Poly (lactic-co-glycolic) acid
PM	Proliferative medium
*POSTN*	Periostin
RNA	Ribonucleic acid
*RUNX-2*	Runt-related transcription Factor
XTT	Cell proliferation kit II (XTT)
